# Single, immediate postoperative instillation of chemotherapy in non-muscle invasive bladder cancer: a systematic review and network meta-analysis of randomized clinical trials using different drugs

**DOI:** 10.18632/oncotarget.9991

**Published:** 2016-06-14

**Authors:** Minyong Kang, Chang Wook Jeong, Cheol Kwak, Hyeon Hoe Kim, Ja Hyeon Ku

**Affiliations:** ^1^ Department of Urology, Seoul National University Hospital, Seoul, Republic of Korea

**Keywords:** urinary bladder neoplasm, chemotherapy, drug therapy, single instillation, systematic review

## Abstract

We performed a network meta-analysis of randomized controlled trials (RCTs) to compare the efficacy of several intravesical chemotherapeutic (IVC) agents after transurethral resection of bladder tumor (TURB) in non-muscle invasive bladder cancer patients. The literature search was conducted using the Embase, Scopus and PubMed databases for RCTs, including patients with single or multiple, primary or recurrent stage Ta or T1 urothelial carcinoma of the bladder managed with a single, immediate instillation of IVC after TURB. Thirteen RCTs met the eligibility criteria. Pair-wise meta-analysis (direct comparison) showed that pirarubicin [hazard ratio (HR): 0.31], epirubicin (HR: 0.62), and MMC (HR: 0.40) were the most effective drugs for reducing tumor recurrence. Bayesian network meta-analysis (indirect comparison) revealed that treatment with pirarubicin (HR: 0.31), MMC (HR: 0.44), or epirubicin (HR: 0.60) was associated with prolonged recurrence-free survival. Among the drugs examined, only pirarubicin reduced disease progression compared to controls. These results suggest that a single, immediate administration of IVC with pirarubicin, MMC, or epirubicin is associated with prolonged recurrence-free survival following TURB in non-muscle invasive bladder cancer patients, though only pirarubicin also reduced disease progression.

## INTRODUCTION

Bladder cancer, the incidence of which has increased over the last decade, is the fourth most common malignancy in men and the eighth most common in women, with 429,000 new cases and 165,000 deaths reported worldwide in 2012; it is also the second most common cause of death due to urological cancer [1]. Approximately 80% of patients with urothelial carcinoma of the bladder initially present with non-muscle invasive disease. Because non-muscle invasive bladder cancers (NMIBCs) may recur and progress to muscle-invasive disease after initial treatment [2], it is necessary to develop efficient therapeutic strategies that reduce recurrence and/or progression. Instillation of intravesical chemotherapy (IVC) is generally recommended immediately after complete transurethral resection of bladder tumor (TURB), especially in patients with low- or intermediate-risk NMIBC or with small-volume, low-grade Ta NMIBC based on European Association of Urology (EAU) and American Urological Association (AUA) guidelines, respectively [3, 4].

Despite accumulating evidence that post-TUR IVC instillation reduces disease recurrence, many clinicians are still reluctant to apply this intervention in patients with NMIBC due to the cost, postoperative care requirements, and unexpected side effects, such as irritative lower urinary tract symptoms, rare hypersensitivity reactions, and extravasation of IVC agents [5, 6]. Moreover, although there are various chemotherapeutic agents for IVC, including mitomycin C (MMC), epirubicin, and gemcitabine, there is currently no consensus regarding which agent produces the best oncological outcomes. Previous studies have primarily focused on the clinical benefits of postoperative IVC in general rather than comparing outcomes associated with different drugs. Various IVC agents may have differing effects on oncological outcomes in NMIBC patients who receive a single, immediate administration regimen following TURB.

To help improve decisions regarding NMIBC treatments, we sought to determine which chemotherapeutic agent is most beneficial as a single, immediate postoperative IVC following TURB by performing a systematic review and network meta-analysis of updated randomized controlled trials (RCTs).

## RESULTS

### Literature search results

**Figure 1:**
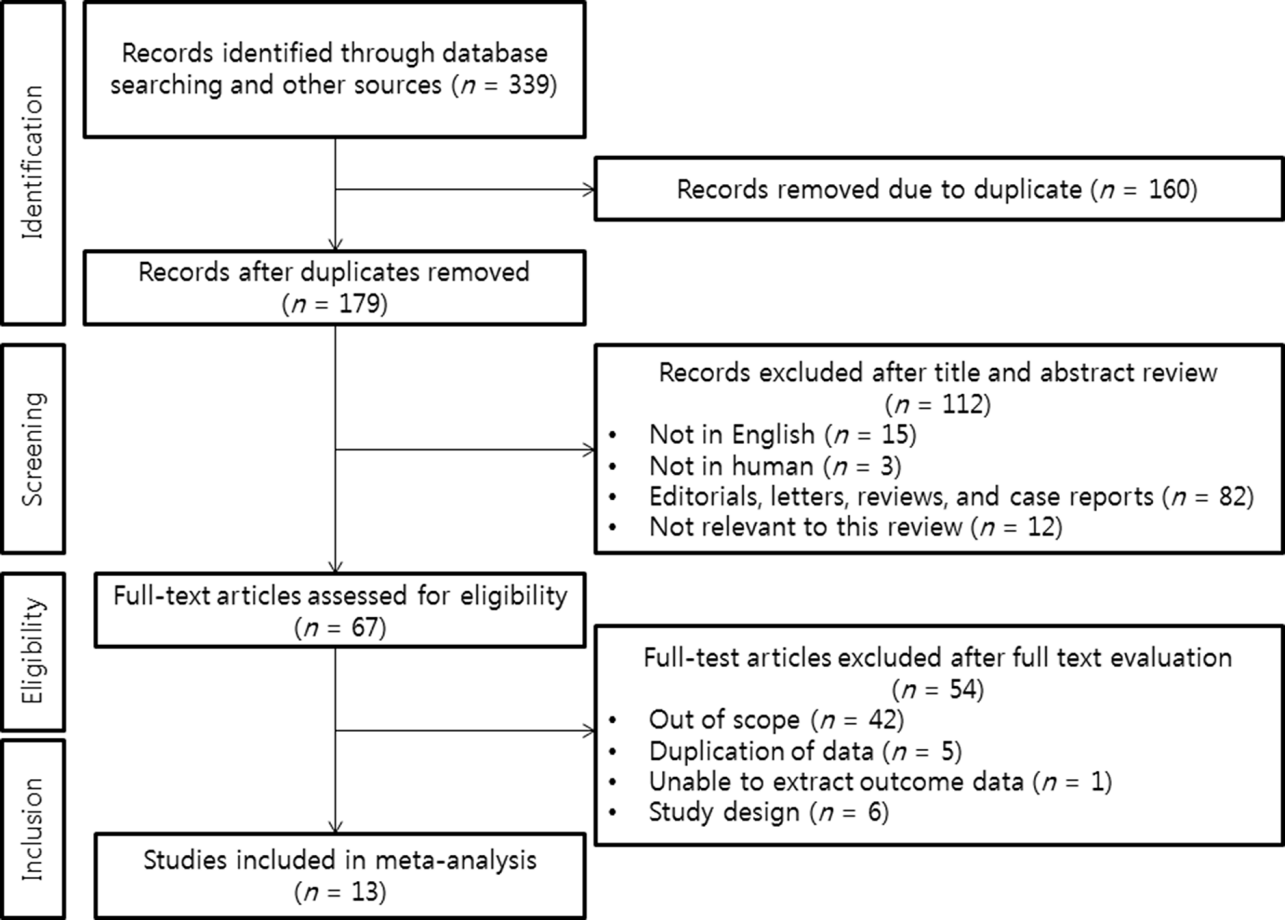
PRISMA statement flow diagram illustrating the search strategy used for the network meta-analysis

Searches yielded 339 potentially relevant studies. We excluded 326 reports that did not meet eligibility criteria. Overall, we included 13 RCTs conducted between 1993 and 2011 in the multiple-treatments meta-analysis. The PRISMA statement flow diagram illustrating the search strategy is shown in Figure 1.

### Overview of included studies

**Figure 2:**
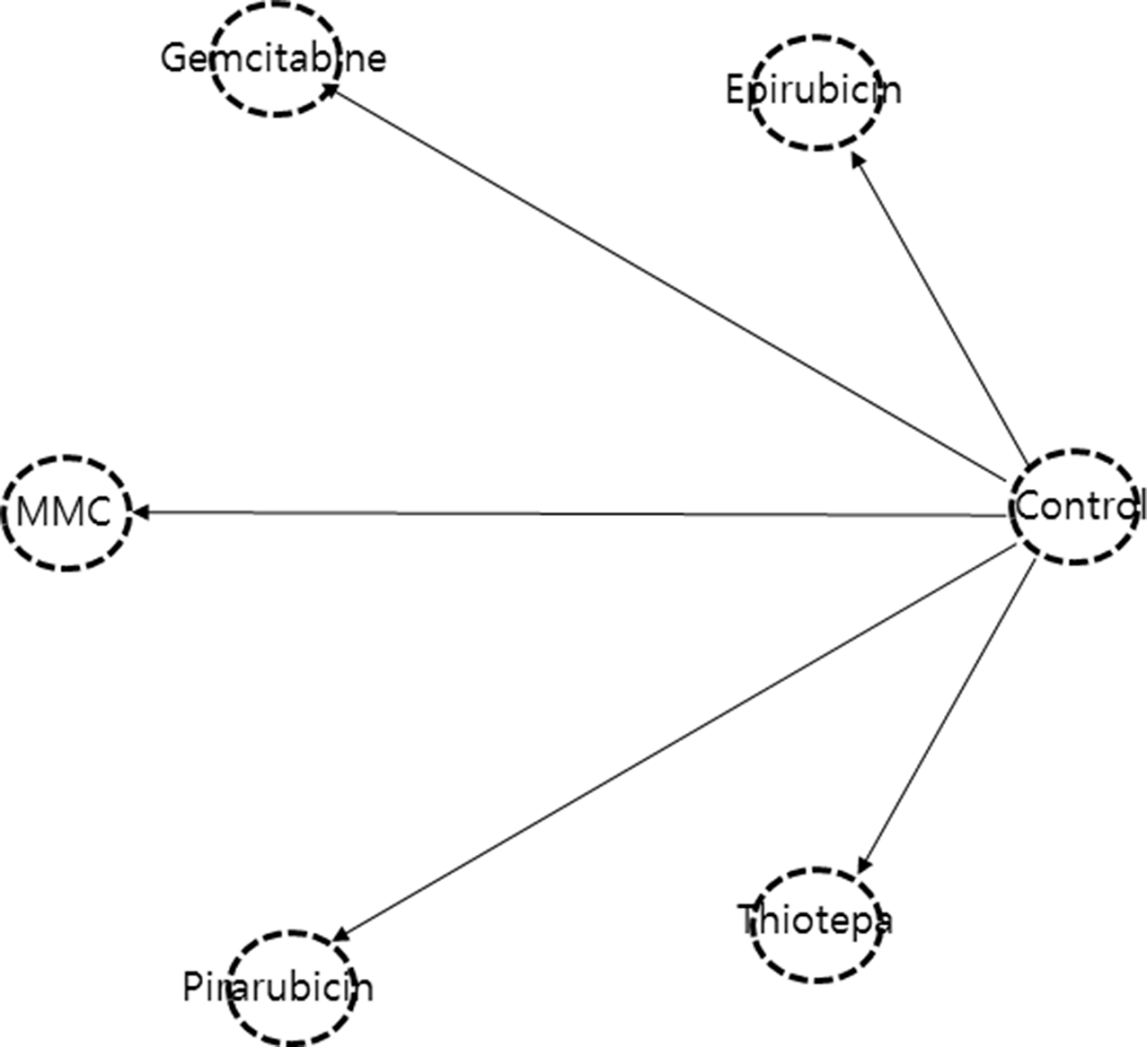
Network geometry of clinical trials of a single, immediate postoperative instillation of chemotherapy for recurrence-free survival in non-muscle invasive bladder cancer Lines represent direct comparison trials.

Figure 2 shows the network of eligible comparisons for the multiple-treatments meta-analysis. Network nodes that are not well-connected should be interpreted with caution.

Detailed characteristics of all studies included in the meta-analysis are listed in Table 1 [7–19]. Five were single-center studies [10, 11, 14, 15, 19] and eight were multicenter studies (one multinational) [7–9, 12, 13, 16–19]. Epirubicin was used in five studies [7, 10, 13, 16, 18], MMC in five studies [9, 11, 14, 15, 19], gemcitabine in one study [17], pirarubicin in one study [12], and thiotepa in one study [8]. In four studies, the control group received an immediate instillation of sterile water or saline after TURB [7, 14, 17, 18]. In all studies, the instillation was given within 24 hours after TURB. Study designs were similar enough to justify aggregating the data for meta-analysis.

**Table 1:** Characteristics of eligible studies

Study number	Author [Reference]	Year	Country	No. of centers	Recruitmentperiod	No. randomized	No. eligible	Chemotherapy	Control group	Irrigation after TURB
1	Oosterlinck [7]	1993	Multination	Multicenter	1986–1989	512	420	Epirubicin 80 mg/50 ml	Sterile water	Yes
2	MRC [8]	1994	UK	Multicenter	1981–1984	281	256	Thiotepa 30 mg/50 ml	TURB alone	NA
3	Tolley [9]	1996	UK	Multicenter	1984–1986	338	306	Mitomycin C 40 mg/40 ml	TURB alone	NA
4	Ali-el-Dein [10]	1997	Egypt	Single	1992–1996	120	109	Epirubicin 50 mg/50 ml	TURB alone	NA
5	Solsona [11]	1999	Spain	Single	1988–1992	131	121	Mitomycin C 30 mg/50 ml	TURB alone	Yes
6	Okamura [12]	2002	Japan	Multicenter	1994–1998	170	160	Pirarubicin 30 mg/30 ml	TURB alone	NA
7	Rajala [13]	2002	Finland	Multicenter	1991–1994	189	134	Epirubicin 100 mg/100 ml	TURB alone	No
8	Barghi [14]	2006	Iran	Single	2003–2005	56	43	Mitomycin C 30 mg/30 ml	Distilled water	Yes
9	El-Ghobashy [15]	2007	Egypt	Single	2002–2005	NA	63	Mitomycin C 30 mg/50 ml	TURB alone	Some
10	Berrum-Svennung [16]	2008	Sweden	Multicenter	1998–2003	404	307	Epirubicin 50 mg/50 ml	Saline	NA
11	Bohle [17]	2009	Germany	Multicenter	2004–2005	355	248	Gemcitabine 2000 mg/100 ml	Saline	Yes
12	Gudjonsson [18]	2009	Sweden	Multicenter	1997–2004	305	219	Epirubicin 80 mg/50 ml	TURB alone	NA
13	De Nunzio [19]	2011	Italy	Single	2000–2009	210	202	Mitomycin C 40 mg/50 ml	TURB alone	Yes

Table 2 shows patient population characteristics from each study [7–19]. This table provides information on tumor status, number of tumors, clinical stage, and tumor grade upon study entry. Only Ta tumors were included in one trial [19], while only G1/G2 tumors were included in six trials [11, 14–16, 18, 19]. In five trials [8, 9, 13, 14, 19], only primary patients were eligible, while in other five trials [7, 11, 14, 15, 19], only patients with single tumors were enrolled. Patients had tumors less than 3 cm in five trials [11, 14–16, 19].

**Table 2:** Patient characteristics from eligible studies

Study number	Author [Reference]	Median age, range (yrs)	No. of gender (male/female)	Clinical stage (LMP/Ta/T1/Tx)	Tumor grade (G1/G2/G3/Gx)	Tumor type (primary/recurrent)	No. of tumor (single/multiple)	Size of tumor (< 3 cm/= 3 cm/NA)	Median FU, range (mons)
1	Oosterlinck [7]	NA	NA	0/310/109/1	187/186/39/8	328/92	420/0	355/53/12	2*^†^
2	MRC [8]	NA	NA	NA	NA	256/0	NA	NA	8.9*
3	Tolley [9]	NA	NA	0/163/139/4	126/150/28/2	306/0	224/76	NA	NA
4	Ali-el-Dein [10]	55.7* (30–72)	75/34	0/19/90/0	20/59/30/0	60/49	69/40	70/39/0	32.2*
5	Solsona [11]	61*	NA	0/59/62/0	63/58/0/0	107/14	121/0	121/0/0	94
6	Okamura [12]	NA (23–82)	NA	0/151/9/0	77/77/6	152/8	152/8	154/4/0	40.8
7	Rajala [13]	NA	91/43	0/109/25/0	72/46/16/0	134/0	99/35	NA	72 (6–102)
8	Barghi [14]	54.8* (22–83)	34/9	0/31/12/0	39/4/0/0	43/0	43/0	43/0/0	15.7* (9–24)
9	El-Ghobashy [15]	NA	NA	0/31/32/0/	32/31/0/0	NA	63/0	63/0/0	NA
10	Berrum-Svennung [16]	72	226/81	0/257/20/30	NA/NA/0/30	153/154	180/127	307/0/0	NA
11	Bohle [17]	66 (24–89)	198/50	0/181/67/0	123/92/27/6	192/56	132/116	NA	23.6 (0–46)
12	Gudjonsson [18]	71	155/64	2/194/18/15	112/92/0/15	115/104	99/117	NA	3.9^†^
13	De Nunzio [19]	61 (42–78)**	133/69	0/202/0/0	149/53/0/0	202/0	202/0	202/0/0	90 (3–112)**

LMP: low malignant potential, NA: not available, FU: follow-up, MRC: Medical Research Council.

* mean

† years

** interquartile range.

### Pair-wise meta-analysis

**Figure 3:**
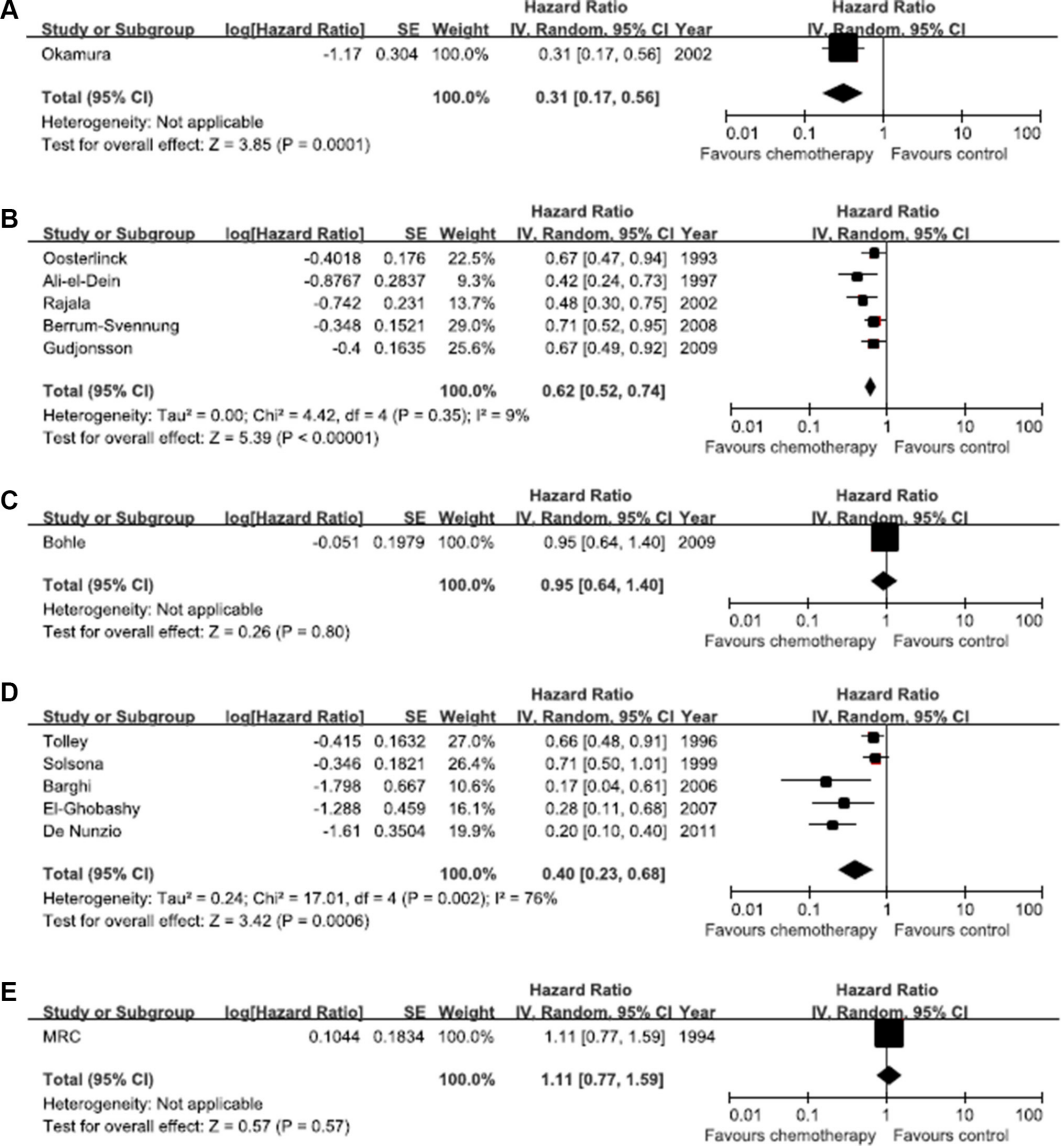
Direct comparisons of efficacy between each pair of chemotherapy treatments The horizontal lines correspond to the study-specific hazard ratio and 95% confidence interval. The area of the squares reflects the study-specific weight. The diamond represents the results for pooled hazard ratio and 95% confidence interval. (**A**) Pirarubicin. (**B**) Epirubicin. (**C**) Gemcitabine. (**D**) Mitomycin (C) (**E**) Thiotepa.

Figure 3 shows the hazard ratios for each direct comparison. There was low heterogeneity among five trials of epirubicin vs. control (*I*^2^ = 9*%, p* = 0.35), while large heterogeneity was observed among trials of MMC vs. control (*I*^2^ = 76*%, p* = 0.002). Because the other comparisons were based on single trials, heterogeneity could not be evaluated. Direct comparisons showed that pirarubicin, epirubicin, and MMC were more effective than control treatment; the effects of gemcitabine and thiotepa in other studies were not statistically significant, as the hazard ratio (HR) 95% (CI) included 1. Funnel plots showed evidence of asymmetry. Egger and Begg test results were significant (all *p* < 0.05) (Supplementary Figure S1), suggesting possible publication bias.

### Bayesian framework network meta-analysis

**Figure 4:**
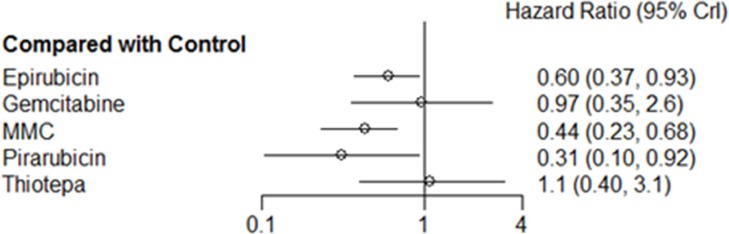
Pooled hazard ratio and 95% credible intervals for recurrence-free survival

**Figure 5:**
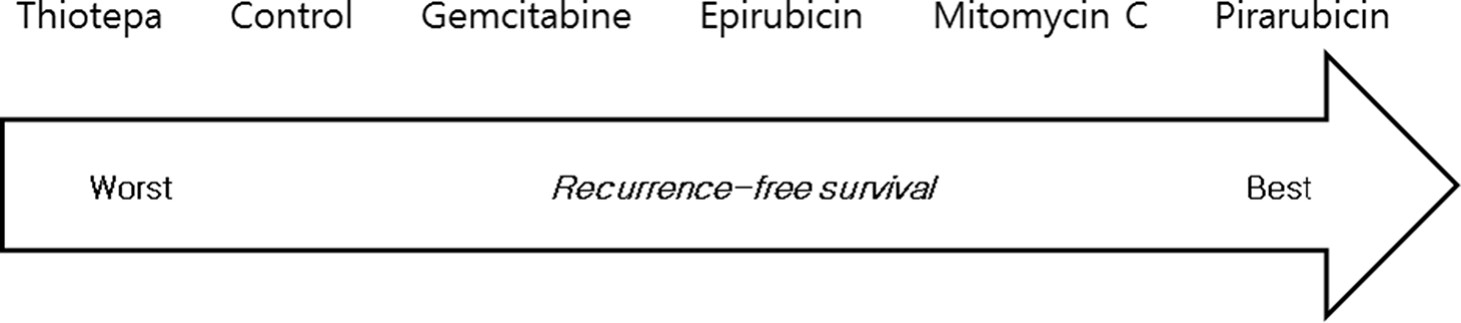
Ranking of treatments in terms of recurrence-free survival benefit Each treatment was ranked using percentages from 2,000 iterations.

A random effects model for a single, immediate postoperative instillation of chemotherapy was selected because the Deviance Information Criteria (DIC) for the random effects model (25.8) was lower than that for the fixed effects model (31.5). Figure 4 shows the results of the network meta-analysis. Treatment with epirubicin (HR: 0.60, 95% credible interval (Crl) 0.37–0.93), MMC (HR: 0.44, 95% Crl: 0.23–0.68), and pirarubicin (HR: 0.31, 95% Crl: 0.10–0.92) were associated with prolonged recurrence-free survival (RFS) compared with controls. For gemcitabine and thiotepa, 95% Crls overlapped the null effect line. Comparison of the results from traditional pairwise meta-analysis and network meta-analysis did not suggest inconsistency between direct and indirect evidence (Supplementary Figure S2). Rankings of the six different treatment strategies (including control) in terms of RFS are summarized in Figure 5, with details provided in Supplementary Table S1. Pirarubicin and MMC were most likely to be ranked the best or the second best, while thiotepa was ranked as the least effective drug.

Bladder cancer progression was reported as a secondary outcome in ten trials [7, 9–12, 14–17, 19]. Supplementary Figure S3 shows the network of eligible comparisons for the network meta-analysis. With the exception of pirarubicin, none of the regimens were more efficacious than controls in reducing progression (Supplementary Figure S4). Comparison of the results from traditional pairwise meta-analysis and network meta-analysis did not suggest inconsistency between direct and indirect evidence (Supplementary Figure S5). Supplementary Figure S6 shows the probability of each specific regimen having a given rank, with details provided in Supplementary Table S2. Pirarubicin usually ranked first, and MMC had a high probability of being ranked second.

## DISCUSSION

Because recurrence rates after TURB are high in NMIBC patients, additional treatments are needed; thus, both EAU and AUA guidelines currently recommend a single, immediate instillation of IVC following TURB in these patients. However, despite promising results in previous RCTs and in our meta-analysis of single-dose IVC in NMIBC patients, its use remains controversial, and post-TUR IVC is often underutilized [5, 20]. For example, Schwartz and colleagues found that post-TUR IVC was administered in only 33–43% of NMIBC patients in European countries [21]. Furthermore, the lack of comparative studies makes it difficult for clinicians to choose the most beneficial agents.

We performed a network meta-analysis to identify the most effective IVC agent for reducing recurrence and progression after TURB in NMIBC patients. To the best of our knowledge, this is the first network meta-analysis based on a Bayesian random effects model focusing primarily on comparing IVC agent efficacy in NMIBC patients undergoing TURB. This statistical technique was developed to compare the relative efficacies of different treatment strategies indirectly when these treatments have not been specifically compared in individual trials [22]. Thus, our network meta-analysis may help assess the efficacy of different IVC agents which have not been compared to each other previously, but which have been compared to similar controls, such as a placebo group.

Notably, our traditional pairwise meta-analysis and network meta-analysis showed that post-TUR IVC instillation was associated with lower rates of bladder tumor recurrence compared to controls. Similarly, De Nunzio *et al*. [23] found that a single, early IVC instillation of mitomycin C reduced the risk of tumor recurrence in low-risk NMIBC. Gudjonsson *et al*. [24] also showed that a single, post-TUR instillation of epirubicin was associated with reduced disease recurrence in 305 NMIBC patients with low- to intermediate-risk bladder tumors. Moreover, Sylvester *et al*. [25] performed the first meta-analysis of this treatment and noted that postoperative IVC resulted in a 11.7% absolute reduction, and a 24.2% relative reduction, in recurrence compared to TUR alone (HR: 0.61, 95% CI: 0.49–0.75) in NMIBC patients. The same group recently reported that a single instillation of post-TUR IVC reduced the relative risk of disease recurrence by 35% (HR: 0.65, 95% CI: 0.58–0.74) and decreased the 5-year recurrence rate from 58.8% to 44.8% [26]. In their evaluation of 18 RCTs that included 3,103 NMIBC patients, Abern and colleagues demonstrated that a single dose of IVC received within 24 hours of resection was associated with a 13% absolute reduction in the risk of tumor recurrence [27].

Conversely, Bohle *et al*. [17] showed that NMIBC patients who received a single, immediate instillation of gemcitabine after TURB had recurrence-free survival rates similar to placebo group patients. Additionally, Berrum and colleagues found that small recurrences prevented by a single instillation of epirubicin could easily be fulgurated in outpatient clinics, indicating that the clinical benefit of post-TUR IVC may be limited [16]. Holmang also suggested that there was no evidence that a single, immediate instillation of IVC after TURB had a beneficial impact on progression and quality of life, although it reduced recurrences of harmless and small tumors compared to TUR alone [28]. Therefore, findings regarding the clinical benefits of a single, immediate postoperative IVC are conflicted.

The other key finding of the present study is the identification of epirubicin (HR: 0.60), MMC (HR: 0.44), and pirarubicin (HR: 0.31) as the most effective IVC agents among those examined, whereas gemcitabine and thiotepa were ineffective in reducing disease recurrence. Recently, in an updated meta-analysis that included 13 RCTs published before 2013, Perlis and colleagues evaluated recurrence-free intervals following treatment with different IVC drugs after TUR. They found that treatment with both MMC (HR: 0.49) and epirubicin (HR: 0.65) were associated with longer recurrence-free interval. Therefore, epirubicin and MMC may be the most effective IVC agents for reducing disease recurrence after TURB, while gemcitabine and thiotepa, which may be relatively ineffective, require further investigation. The recent Cochrane review by Shelley *et al*. also indicated that a single instillation of gemcitabine following TURB was no more effective than placebo in reducing tumor recurrence [29].

Interestingly, only treatment with pirarubicin was associated with reduced disease progression rates; other IVC regimens were no more effective than controls in preventing progression. However, bladder cancer progression was a rare event in the pirarubicin trial, occurring in only 1.5% of controls (1 of 66) and in 0% of patients receiving pirarubicin (0 of 68). Therefore, conclusions drawn from this analysis should be considered with caution.

Several limitations of this study should be addressed in future research. First, as in other meta-analyses, unknown or uncontrolled variables in the included trials affect the results of the current study. Short follow-up periods, inadequate sample sizes, and non-generalizable populations are common limitations of RCTs. Second, network meta-analysis requires combining evidence from studies with markedly different designs [30]. For example, Stettler *et al*. [31] conducted a network meta-analysis to compare the clinical outcomes of drug-eluting and bare-metal stents in patients with coronary artery disease. However, eligibility criteria for percutaneous coronary intervention and the definition of myocardial infarction were not consistent across the included trials. Third, because the current meta-analysis was based on a random effects model, smaller studies may have disproportionately affected our results [32]. Finally, although we demonstrated that post-TUR IVC agents have different efficacies in NMIBC patients following TURB, the potential mechanisms responsible for these differences remain unknown. Nevertheless, these results merit consideration, especially considering that publication bias and heterogeneity between the included studies, which are crucial problems for traditional meta-analyses, were not significant.

In summary, our meta-analysis showed that a single, immediate IVC treatment with pirarubicin, MMC, or epirubicin after TURB was associated with prolonged RFS in NMIBC patients. In contrast, gemcitabine and thiotepa did not reduce disease recurrence compared to controls. However, among the drugs examined, only pirarubicin was effective in preventing disease prevention compared to controls. Although larger RCTs comparing these agents are required in order to provide more direct evidence, our study may aid in the selection of the most appropriate IVC agent following TURB in NMIBC patients.

## MATERIALS AND METHODS

### Search strategy

This review was carried out according to the PRISMA statement [33]. A literature search was performed using Embase, Scopus, and PubMed databases for all RCTs published prior to December 31, 2015. Keywords used were “randomized clinical trial”, “bladder cancer”, “single”, and “intravesical”. In addition to searching the databases, the reference lists of all included studies, meta-analyses, and reviews were manually searched.

The search was restricted to studies published in English. Two investigators (MK and CWJ) independently reviewed the titles, abstracts, and studies to establish whether they met the inclusion criteria. Conflicts between reviewers were resolved by consensus.

### Eligibility criteria

We determined study eligibility according to predefined selection criteria [33]. Prospective RCTs involving patients with single or multiple, primary or recurrent stage Ta or T1 urothelial carcinoma of the bladder managed with a single, immediate instillation of chemotherapy after TURB were included. Patients who were treated with TURB alone or placebo instillation after TURB served as comparators. Disease recurrence was the primary endpoint and progression was the secondary endpoint. Studies that were not randomized or prospective in their design were excluded from the analysis.

Studies involving patients with non-urothelial carcinoma (e.g, squamous, adenocarcinoma) or alternative routes of administration (i.e., intravenous, oral, intramuscular injection) were also excluded. Additionally, studies that did not use controls or that used historical controls were excluded, as were studies in which the outcomes of interest were not reported or were impossible to calculate based on the published results. When reports overlapped or there were duplicates, we retained the data with the longest follow-up period.

### Data extraction and synthesis

Two reviewers (CK and HHK) performed all data extraction, including study characteristics and outcome data. For each study included in the network meta-analysis, the following information was extracted: name of the first author, year of publication, geographic location, period of recruitment, sample size (randomized patients, total and per arm), median age, percentage of patients who showed recurrence and/or progression, and chemotherapy regimen and dosage delivered. Discrepancies were discussed until consensus was reached.

### Data synthesis

To assess the relative effectiveness of each treatment, placebo or TURB alone were considered the reference treatments for direct and indirect analyses, respectively. All treatments using a given drug were considered together, regardless of differences in dosage schemes among studies.

The efficacy of a single, immediate instillation of chemotherapy after TURB was compared to TURB alone with respect to the primary endpoint (recurrence) and the secondary endpoint (progression). For time-to-event comparisons, the starting point was the date of randomization.

### Statistical analysis

We extracted or estimated the logarithm of the hazard ratio (log[HR]) and its variance. We calculated HRs and corresponding 95% CIs to assess the effect of each chemotherapy regimen on outcomes. When HRs and 95% CIs were not available, they were approximated using the methods described by Parmar *et al*. [34]; we imputed HR and its variance using the number of events (E_1_, E_2_) and randomized patients (T_1_, T_2_) in each arm and the presented log-rank *p* value. We estimated the variance of the log(HR) using the formula (T_1_+T_2_)^2^/[(E_1_+E_2_)T_1_T_2_] and then estimated the natural logarithm of the HR such that the *p* value matched that of the log-rank test.

When two or more studies comparing the same regimen were available, a direct meta-analysis was performed using the DerSimonian and Laird random effects model [35]. Exchangeability was assessed by examining heterogeneity in each head-to-head comparison. Between-study heterogeneity was estimated by using the I^2^ statistic; typically, values above 50% indicate high heterogeneity, values from 25–50% indicate moderate heterogeneity, and values below 25% indicate low heterogeneity. Publication bias was assessed by visual inspection of funnel plots as well as with the Egger linear regression test and the Begg rank correlation test. Since data on adverse effects were analyzed in previously published meta-analyses [26, 27, 36], adverse effects were not examined in this analysis.

We performed a network meta-analysis using a random-effects model. Model parameters were estimated using a Markov chain Monte Carlo method called Gibbs sampling, as implemented in WinBUGS 1.4 (MRC Biostatistics Unit, Cambridge, UK) [37]. The selection of a fixed or random effects model for reported outcomes was based on the model fit criteria (Deviance Information Criteria, DIC), which penalizes greater model complexity [22]. Each analysis was based on non-informative priors for effect sizes and precision. To avoid potential selection bias, we incorporated all data presentations in a single analysis using the methods described by Woods *et al*. [38]. The median of the posterior distribution was used as a point estimate of treatment effect. Effect sizes together with 95% credible intervals (Crls) were used to make different comparisons across studies. In the presence of minimally informative priors, Crls can be interpreted similarly to conventional CIs.

We also examined inconsistency between direct and indirect estimates using a modified back-calculation approach [39]. The quality of the models was examined by inspecting convergence using Gelman-Rubin-Brooks plots, assessing autocorrelation between iterations of the Markov chain, and determining whether the MC error was less than 5% of the posterior standard deviation.

Version 5.0 RevMan statistical software (The Cochrane Collaboration, Copenhagen) was employed for the direct meta-analysis. Bayesian framework analyses were performed in R 3.2.2 (R development Core Team, Vienna, http://www.R-project.org) with the GeMTC package. A *p* value less than 0.05 was considered statistically significant. Unless otherwise stated, all *p* values were two-sided.

## SUPPLEMENTARY MATERIALS FIGURES AND TABLES


