# Supplementary material for: Single, immediate postoperative instillation of chemotherapy in non-muscle invasive bladder cancer: a systematic review and network meta-analysis of randomized clinical trials using different drugs
Source: Oncotarget. 2016 Jun 14;7(29):45479–88. doi: 10.18632/oncotarget.9991 (PMC5216735; doi:10.18632/oncotarget.9991)
Supplement: Supplementary file 1 [file oncotarget-07-45479-s001.pdf]

Single, immediate postoperative instillation of chemotherapy in non-muscle invasive bladder cancer: A systematic review and network meta-analysis of randomized clinical trials using different drugs

Supplementary Materials

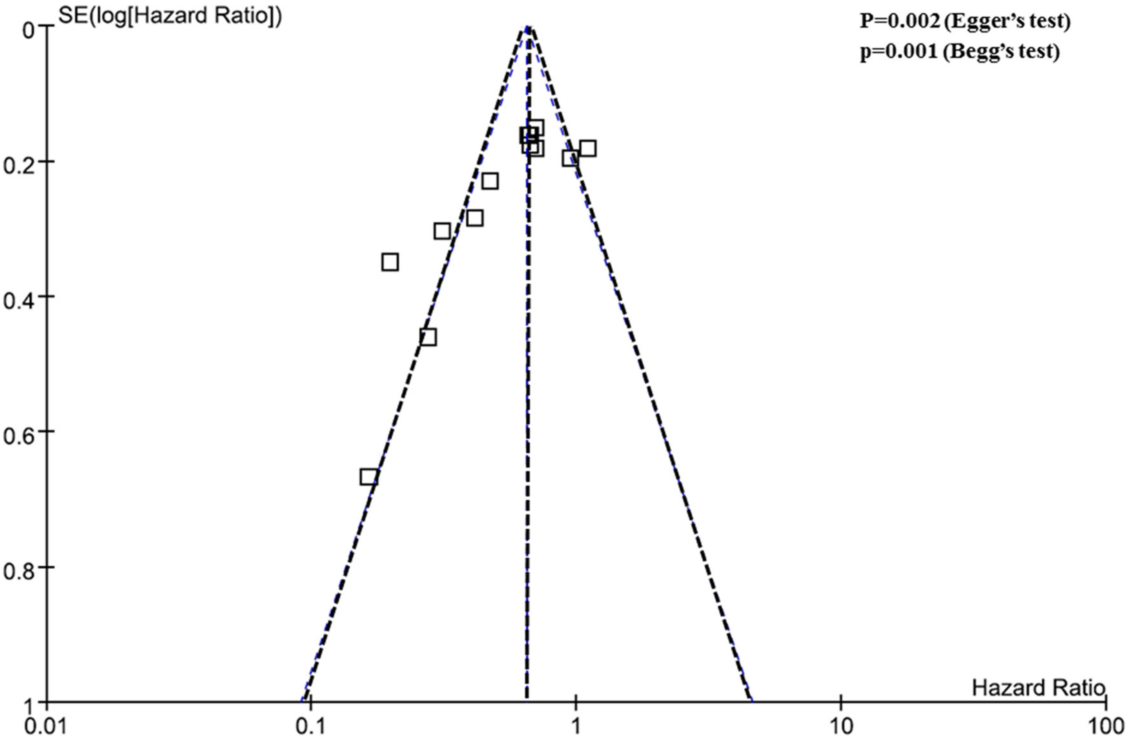

Supplementary Figure S1: Funnel plots for publication bias test. Each point represents a separate study of the indicated association. Vertical lines represent mean effect sizes.

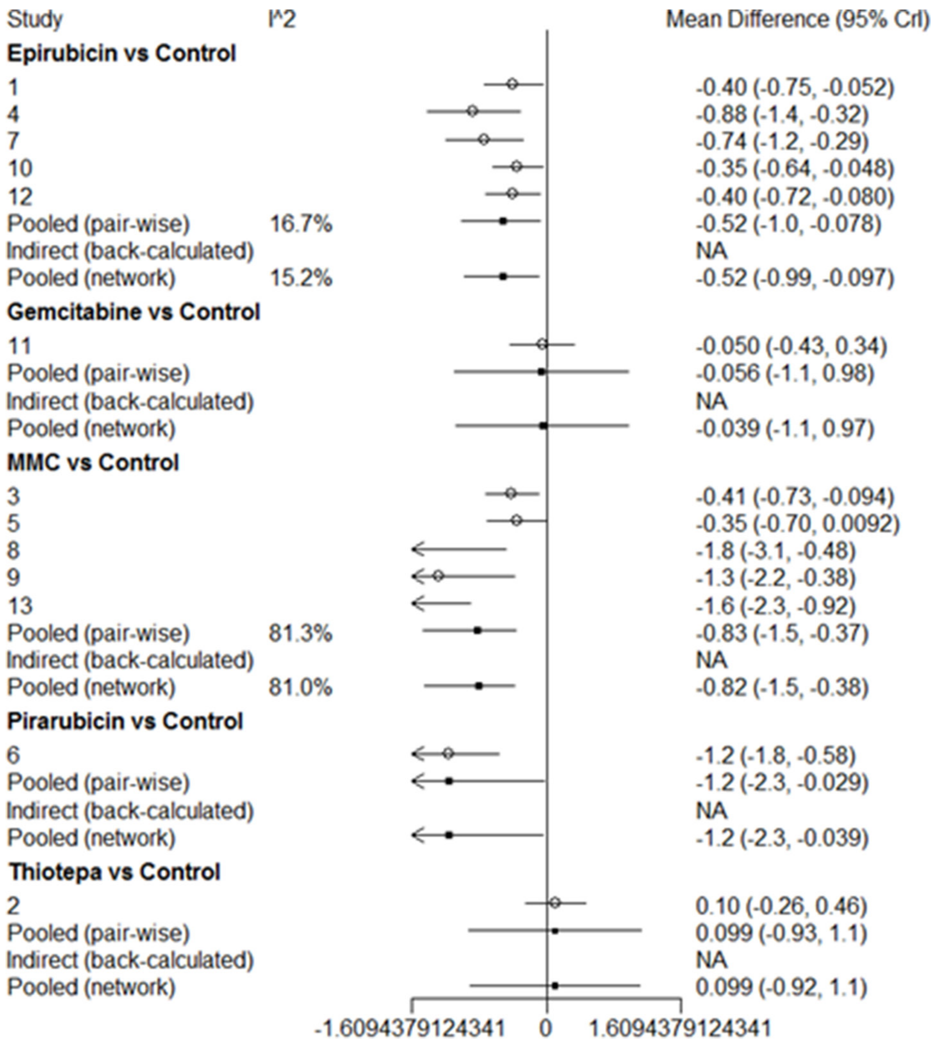

Supplementary Figure S2: Inconsistency test for recurrence-free survival.

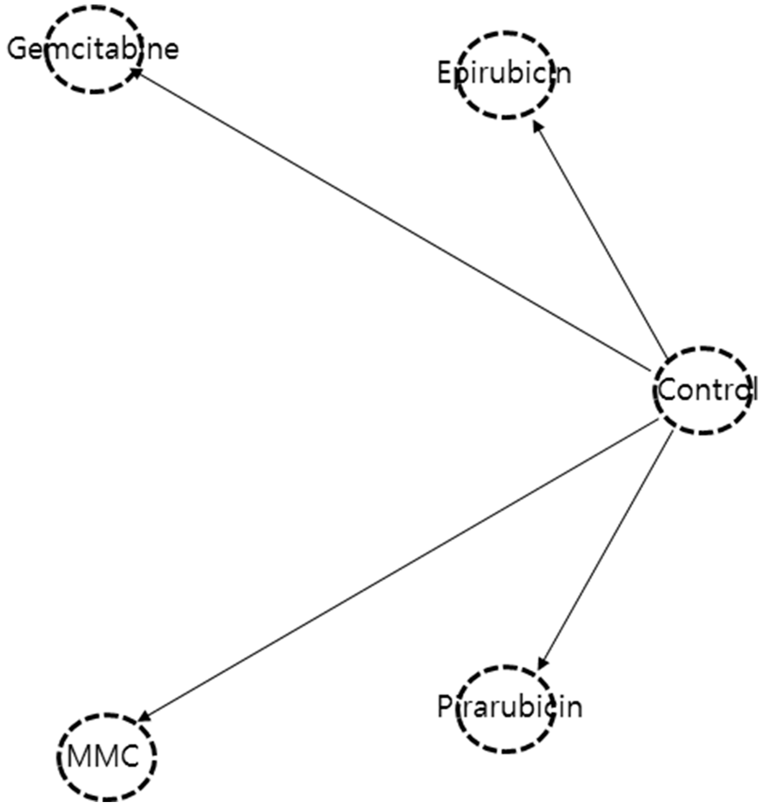

Supplementary Figure S3: Network geometry of clinical trials of a single, immediate postoperative instillation of chemotherapy for progression-free survival in non-muscle invasive bladder cancer. Lines indicate direct comparison trials.

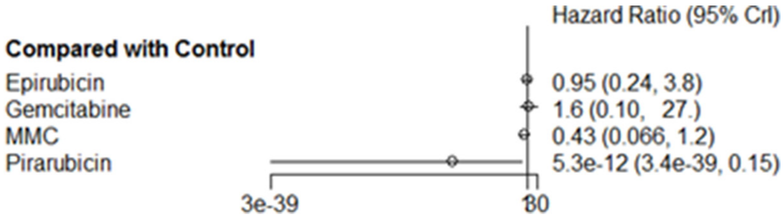

Supplementary Figure S4: Pooled hazard ratios and 95% credible intervals for progression-free survival.

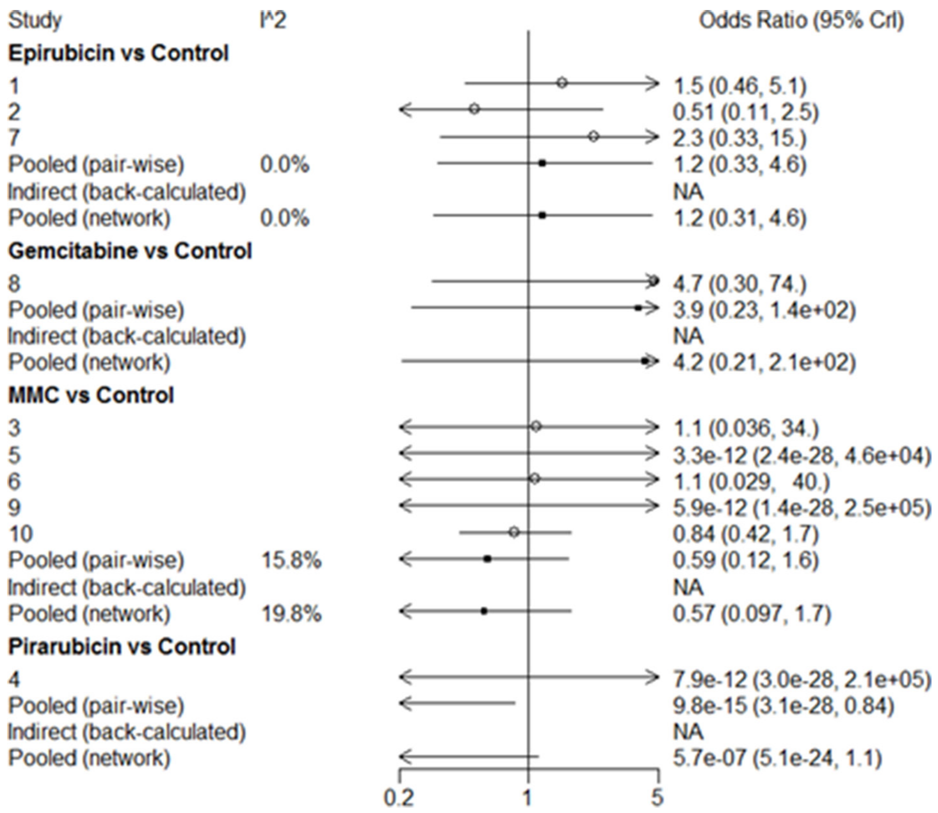

Supplementary Figure S5: Inconsistency test for progression-free survival.

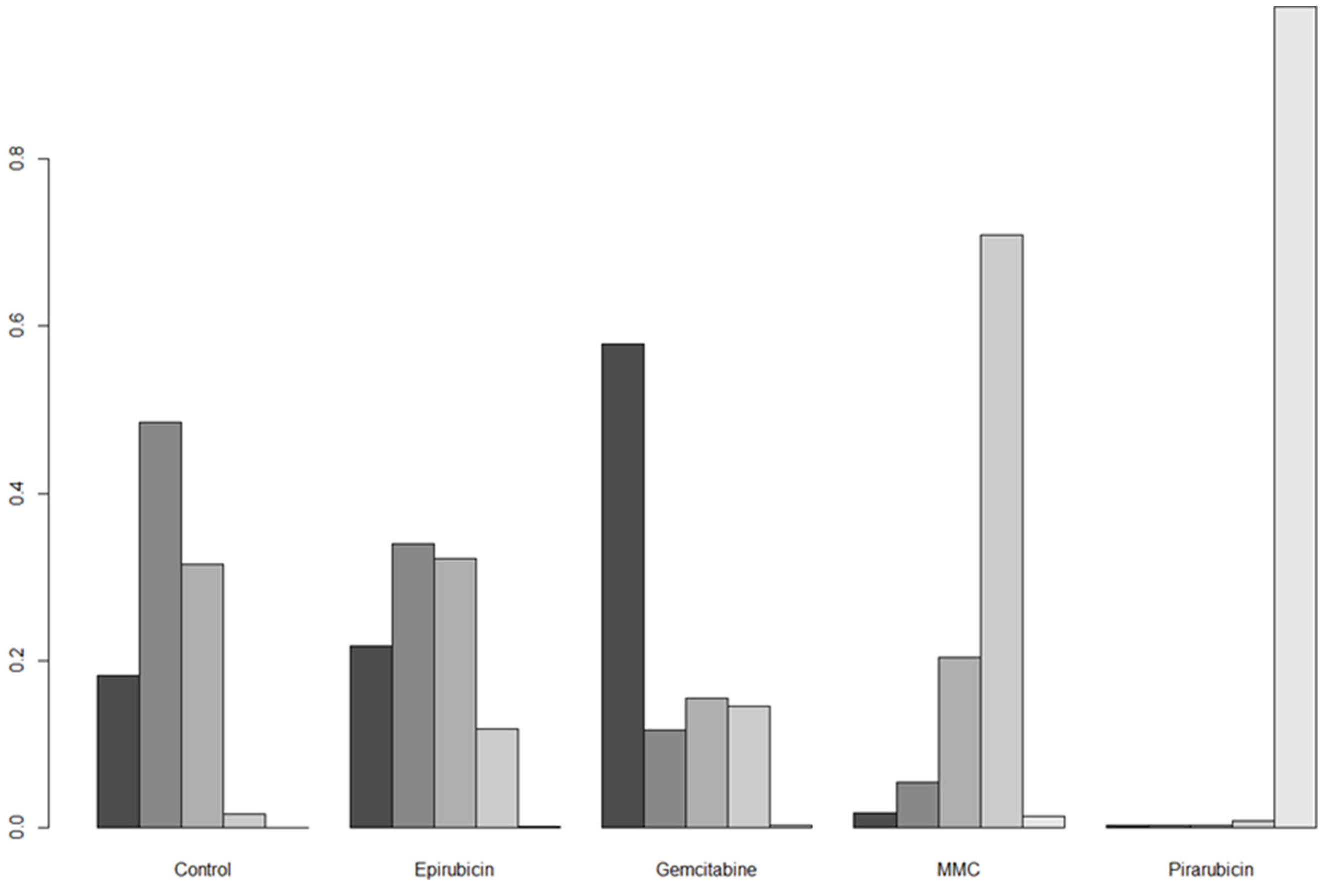

Supplementary Figure S6: Rankograms for the single, immediate postoperative instillation of chemotherapy network. The size of each bar corresponds to the probability that each treatment is assigned that specific rank.

Supplementary Table S1: Ranking of recurrence-free survival (% of 2,000 iterations)

Rank	Thiotepa	Control	Gemcitabine	Epirubicin	Mitomycin C	Pirarubicin
1	1.2	0.0	1.8	2.7	23.4	70.8
2	2.7	0.1	4.1	17.1	59.8	16.3
3	6.6	1.1	9.9	60.5	14.0	8.0
4	20.3	28.2	29.8	16.5	2.4	2.9
5	23.0	49.5	23.3	2.8	0.3	0.1
6	46.4	21.3	31.1	0.4	0.1	0.1

Lower rank indicates greater survival benefit.

Supplementary Table S2: Ranking of progression-free survival (% of 2,000 iterations)

Rank	Gemcitabine	Control	Epirubicin	Mitomycin C	Pirarubicin
1	0.3	0.0	0.1	1.4	98.1
2	14.6	1.7	11.9	71.0	0.9
3	15.6	31.5	32.2	20.4	0.3
4	11.7	48.5	34.0	5.4	0.3
5	57.9	18.3	21.8	1.8	0.3

Lower rank indicates greater survival benefit.
